# Genome-Wide Characterization and Expression Analysis of NHX Gene Family under Salinity Stress in *Gossypium barbadense* and Its Comparison with *Gossypium hirsutum*

**DOI:** 10.3390/genes11070803

**Published:** 2020-07-16

**Authors:** Umar Akram, Yuhan Song, Chengzhen Liang, Muhammad Ali Abid, Muhammad Askari, Aye Aye Myat, Mubashir Abbas, Waqas Malik, Zulfiqar Ali, Sandui Guo, Rui Zhang, Zhigang Meng

**Affiliations:** 1 Biotechnology Research Institute, Chinese Academy of Agricultural Sciences, Beijing 100081, China; umar02kwl@gmail.com (U.A.); m13224233029@163.com (Y.S.); liangchengzhen@caas.cn (C.L.); ali_abid0344@hotmail.com (M.A.A.); Askaricaas5@yahoo.com (M.A.); aamyat19may@gmail.com (A.A.M.); Mubashirabbas3164@yahoo.com (M.A.); guosandui@caas.cn (S.G.); zhangrui@caas.cn (R.Z.); 2 Genomics Lab, Department of Plant Breeding and Genetics, Faculty of Agricultural Science & Technology, Bahauddin Zakariya University, Multan 60000, Pakistan; waqasmalik@bzu.edu.pk; 3 Institute of Plant Breeding and Biotechnology, MNS-University of Agriculture, Multan 60000, Pakistan; zulfiqarpbg@hotmail.com

**Keywords:** *G. barbadense*, *GbNHX*, phylogenetic, abiotic stress, Na^+^/H^+^ antiporter, amiloride, *Cis*-elements

## Abstract

Cotton is an important economic crop affected by different abiotic stresses at different developmental stages. Salinity limits the growth and productivity of crops worldwide. Na^+^/H^+^ antiporters play a key role during the plant development and in its tolerance to salt stress. The aim of the present study was a genome-wide characterization and expression pattern analysis under the salinity stress of the sodium-proton antiporter (NHX) of *Gossypium barbadense* in comparison with *Gossypium hirsutum*. In *G. barbadense*, 25 *NHX* genes were identified on the basis of the Na^+^_H^+^ exchanger domain. All except one of the *G. barbadense* *NHX* transporters have an Amiloride motif that is a known inhibitor of Na^+^ ions in plants. A phylogenetic analysis inferred three classes of *GbNHX* genes—viz., Vac (*GbNHX1*, *2* and *4*), Endo (*GbNHX6*), and PM (*GbNHX7*). A high number of the stress-related *cis*-acting elements observed in promoters show their role in tolerance against abiotic stresses. The Ka/Ks values show that the majority of *GbNHX* genes are subjected to strong purifying selection under the course of evolution. To study the functional divergence of *G. barbadense* *NHX* transporters, the real-time gene expression was analyzed under salt stress in the root, stem, and leaf tissues. In *G. barbadense*, the expression was higher in the stem, while in *G. hirsutum* the leaf and root showed a high expression. Moreover, our results revealed that *NHX2* homologues in both species have a high expression under salinity stress at higher time intervals, followed by *NHX7*. The protein-protein prediction study revealed that *GbNHX7* is involved in the CBL-CIPK protein interaction pathway. Our study also provided valuable information explaining the molecular mechanism of Na^+^ transport for the further functional study of Gossypium *NHX* genes.

## 1. Introduction

Soil salinity is one of major abiotic stresses that limits crop production worldwide [1], with an estimated 45 million hectares of irrigated land reported to be under salinity stress. The world’s food production is mainly dependent on irrigated land, as it produces twice as much as the rain-fed area, therefore high salinity is a threat to sustainable crop production for the ever-increasing population [2,3]. By the year 2050, about 50% of all cultivable land is predicted to be affected by high salinization [4,5].

Most plants, being glycophytes, are affected by high levels of salt in the soil [6]. Plants have developed different mechanisms such as ionic stress pathways, oxidative stress pathways, and detoxification signalling to cope with the high soil salinity and toxicity of Na^+^ and Cl^−^ ions [7]. Many cellular processes conferring stress tolerance and regulating plant growth and development are dependent upon pH and ion homeostasis [8]. Ion-specific salinity is caused by the accumulation of toxic concentrations of sodium (Na^+^) and/or chloride (Cl^−^) ions, especially in the older leaves [9]. In most plant species, the Na^+^ reaches the toxic concentration earlier than other salts [10]. Two non-selective cation channels (NSCC) are the major source of entry of Na^+^ into the cell; voltage-dependent and voltage-independent cation channels. The voltage-independent cation channels are thought to be a significant way of entering for Na^+^ ions. [11,12]. Sodium-hydrogen antiporters (*NHX*) are important antiporter genes which can help plants to exclude Na^+^ and Cl^-^ ions through membranes or deposits in the vacuole to maintain the cell osmotic level [13]. Vacuole-bounded *NHX* antiporters regulate pH by countering acidity due to H^+^ pumps and functions such as H^+^ leaks to maintain the pH [14]. Besides the compartmentalization of Na^+^, *NHX*s could play a role in increasing the salinity tolerance by adjusting the K^+^ homeostasis [15,16,17].

Sodium-hydrogen antiporters (*NHX*) belongs to the cation proton antiporter1 (CPA1) family, which seems to have evolved from the sodium-proton antiporter (NhaP) genes in prokaryotes [18,19,20]. Human *HsNHE* was the first eukaryotic sodium hydrogen exchanger gene to be identified [21]. Meanwhile, in plants *NHX1* was the first sodium hydrogen exchanger identified in Arabidopsis [22]. Besides contributing to salt tolerance [23], *NHX*s have diverse roles in biochemical and physiological processes, which include maintaining the pH in flowers [24], cellular expansion [25], K^+^ homeostasis [26], protein targeting, and vesicular trafficking [19,27,28]. Arabidopsis have eight members of the *NHX* genes that are further categorised into three groups based on their location. *AtNHX1-4* is located in the vacuolar membrane, *AtNHX7* and *AtNHX8* are located in the plasma membrane, while *AtNHX5* and *AtNHX6* are located in the endosomal compartments [29,30]. The plasma membrane-bounded activity of the Na^+^/H^+^ antiporter activity has been studied in barley [31], tomato [32], and wheat [33], while a tonoplast-associated Na^+^/H^+^-antiporter activity has been reported for sugar beet [34], barley [35], sunflower [36], and Arabidopsis [23]. In Arabidopsis, the Na^+^ ion efflux is processed by the plasma membrane located Na^+^/H^+^ antiporter *AtSOS1* under high salinity [37], while the vacuolar Na^+^/H^+^ antiporter catalyzes the sequestration of Na^+^ in vacuoles. Different studies have shown that the over-expression of *NHX1* enhanced the plant tolerance towards salinity in different crops [20,23,38,39,40]; wheat *NHX2* (*TaNHX*) transformed into alfalfa enhanced the salinity tolerance due to the homeostasis of potassium [41], whereas the *nhx5 nhx6* double-knockout mutant in Arabidopsis aborted the transport through the tonoplast, increasing the sensitivity to salt stress [29]. These studies provide convincing proof of the involvement of the *NHX* genes in salinity tolerance, and this can be further explored in economically important crops.

Cotton is a worldwide leading textile fiber crop that has a significant impact on the economy of many agricultural-based countries [42]. *G. barbadense* and *G. hirsutum*, the two allotetraploids, are the most widely cultivated cotton species. With drastic environmental changes leading to a decline in the cultivated land area, like many other economic crops, cotton planting fields are moving to salinity and drought-affected areas. Overall, cotton crop production is always hindered by abiotic stresses, such as cold, heat, drought, and salinity [43,44]. Despite the fact that there are some natural varieties that are tolerant to drought and salinity, most high-quality cotton cultivars are sensitive to drought and salinity; in those cultivars, high soil salt concentrations affect the germination and emergence of seedlings [45,46], root growth [47,48], flowering, boll development, and fiber quality [49,50,51], causing an up to 50% loss in yield [52]. Finding the mechanism of abiotic stress tolerance will be of great significance for cotton production and genetic improvement.

In this study, we performed a genome-wide analysis of *NHX* genes in *G. barbadense* in comparison with *G. hirsutum*, including the phylogenetic relationships, a motif analysis, promoter analysis, the transcript expression under salt stress in different tissues, the chromosomal location, and the gene structures. The sequencing of many cotton species provides a wide range of genome data resources for gene family research [53,54,55,56,57,58]. Through a systematic analysis of all the members of the *NHX* gene, we can compare the gene regulation, expression pattern, and eventually their biological functions in cotton.

## 2. Materials and Methods

### 2.1. Characterization of Sodium Proton Antiporters

The *NHX* transporters are characterized by an Na^+^_H^+^_Exchanger domain (PF00999) (http://pfam.xfam.org/) [59]. The amino acid sequences of the *NHX* genes of *G. hirsutum* (JGI Version 2.0), *G. barbadense* (HAU, Version 1.0), *G. arboreum* (CRI, Version 1.0), and *G. raimondi* (JGI, Version 2.0) were downloaded from CottonFGD (https://cottonfgd.org/) [60] and were scanned against the Na^+^_H^+^_Exchanger domain using the HMMER 3.1b2 online software (https://www.ebi.ac.uk/Tools/hmmer/) [61]. The transmembrane domain prediction was made using the TMHMM server v.2.0 (http://www.cbs.dtu.dk/services/TMHMM/) [62], and, for the subcellular localization, CELLO v.2.5 (http://cello.life.nctu.edu.tw/) [63,64] was used. Netphos 3.1 [65] was used for the phosphorylation sites, and the prediction of the conserved motifs was carried out by MEME [66] with parameters such as the 2–20 motif sites, 10 no. of motifs, and 6–50-wide motif width; for all other tools, the default settings were used. The molecular weight (MW) and isoelectric point (pI) of the amino acid sequences were predicted using the online program ProtParam (http://web.expasy.org/protparam/).

### 2.2. Phylogeny and Divergence Analysis

A maximum likelihood phylogenetic tree was constructed with the amino acid sequences of *Gossypium hirsutum* (Gh), *Gossypium barbadence* (Gb), *Gossypium arboreum* (Ga), *Gossypium raimondii* (Gr), *Arabidopsis thaliana* (At), *Vitis vinifera* (Vv), *Poplus trichocarpa* (Ptr), *Sorghum bicolor* (Sb), *Medicago truncatula* (Mt), *Eutrema halophilum* (Eh), and *Physcomitrella patens* (Pp). The *NHX* protein sequences of four cotton species were downloaded from Cotton FGD and were already reported for *S. bicolor* and *P. patens* [67]. *T. halophilum*, also known as *E. halophilum* [68]; *V. vinifera*; *P. trichocarpa* [69]; *A. thaliana* [37]; and *M. truncatula* [70] were downloaded from the online Phytozome v11 (https://phytozome.jgi.doe.gov/pz/portal.html) (Table S1). All the retrieved amino acid sequences were confirmed with the hidden Markov model (HMM) using the PF00999 Na^+^_H^+^ exchanger domain. The sequences were then aligned using muscle and subjected to a phylogenetic analysis using MEGA 10.0 [71]; the bootstrap value was kept at 1000. The resulting tree was visualized using iTOL v5 (https://itol.embl.de/) [72]. Tbtools [73] were used to estimate the gene duplication events. To further calculate the synonymous (d_s_) and non-synonymous (d_N_) substitution rates, the PAL2NAL program [74] was used.

### 2.3. Promoter and Gene Structure Analysis

The upstream 2 Kb sequences for all the *NHX* genes of *G. barbadense* and *G. hirsutum* were analyzed in silico to find out the potential *Cis*-acting elements. All the promoters were submitted to PLANTCARE [75], and the resulting *Cis*-acting elements were categorized based on their functional class. The Gene Structure Display Server tool was used for the analysis of the gene structure [76].

### 2.4. Protein-Protein Interaction and Physical Mapping

The STRING database (https://string-db.org) was used to predict the protein-protein interactions. The genomic coordinates of the transporters were extracted from the Cotton FGD Database (https://cottonfgd.org/) [60] using the HAU assembly for *G. barbadense* and the JGI assembly for *G. hirsutum* and then used to map the genes onto different chromosomes physically.

### 2.5. Expression Analysis under Salinity

To investigate the expression level of *NHX* transporters under salinity stress, the *G. barbadense* cultivar Hai7124 and the *G. hirsutum* cultivar TM-1 were sown in pots in greenhouse conditions with temperatures ranging from 25 to 30 °C and with 12 h light and 12 h dark. At the emergence of true leaves, the seedlings were treated with a 400 mM salinity level and tap water served as a control. Samples were taken from the leaves, stems, and roots 0 h, 3 h, 6h, and 12 h after the treatment, then they were snap-frozen in liquid nitrogen and subsequently stored at −80 °C until the RNA was extracted.

### 2.6. RNA Extraction and Quantitative Real-Time PCR Analysis

The total RNA was isolated from all the samples using the EASYspin RNA plant-kit (Cat#DR103-03) following the instruction manual. DNaseI (RNase-free) was used to eliminate the genomic DNA contamination in the RNA samples. The concentration and purity was checked by Thermo fisher Scientific Nano-Drop One and run on 1% agarose gel. The total RNA (5 g) was taken as a template for a first strand cDNA synthesis using the iScriptTm Reverse Transcription Supermix for RT-qPCR (BIO-RAD, Hercules, CA, USA).

BIO-RAD’s CFX Connect Real-Time PCR Detection System was used to study the relative expression level of the *G.barbadense* and *G. hirsutum* NHX genes using the iTAQ UNIVERSAL SYBR GREEN MIX (BIO-RAD) with gene-specific primers. Each gene expression was normalized with the Actin genes [77]. The thermal cycler conditions were 95 °C for 3 min, followed by 40 cycles of 95 °C for 10 s, 60 °C for 1 s, and 72 °C for 30 s, and the melting curve stage was at 95 °C for 10 s, 65 °C for 1 min, and 97 °C for 1–5 s.

## 3. Results

### 3.1. Characterization of NHX Genes in Cotton Species

**Figure 1:**
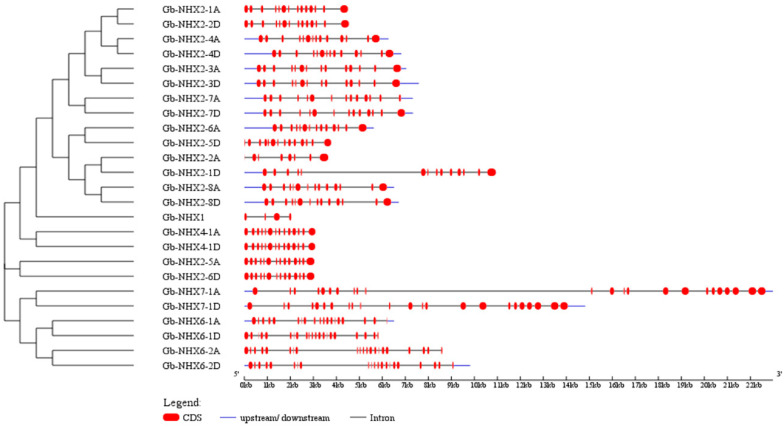
Gene Structure of the *G. barbadense NHX* transporters. Red box represents the exons and the black lines represent the introns.

To retrieve the members of the *NHX* gene family, we searched four cotton species’ genome data based on the Na+_H+_Exchanger domain (PF00999). A total of 25 *NHX* genes in *G. barbadense*, 23 in *G. hirsutum*, 13 in *G. arboreum*, and 13 in *G. raimondii* were identified. We further determined the biophysical properties of the *G. barbadense NHX* genes including the locus ID, CDS length (bp), protein length (aa), Na^+^/H^+^ exchanger domain, predicted protein molecular weight (MW), predicted cellular localization, isoelectric points (pI), and trans-membrane domains. The *NHX* proteins were predicted to be localized on the plasma membrane, endoplasmic reticulum, and vacuole with number of amino acids ranging from 164 to 1152. The molecular weight ranges from 18.66 kDa (*Gb-NHX1*) to 128.14 kDa (*Gb-NHX7-1D*) (Table 1, Figure S1). Previously, it has been reported that the distribution pattern of intron/exon in a gene play a vital role in its biological function. The number of exons for both the G. *barbadense* and G. *hirsutum* transporters varies from 14 to 23, with the exception of *NHX1* (Figure 1). Moreover, the gene structure analysis of the tetraploid cotton species (*G. hirsutum* and *G. barbadense*) along with the phylogeny results showed that the genes with a similar intron/exon pattern clustered near to each other in same groups (Figure S2). An in silico analysis revealed that the *NHX* transporters are mostly phosphorylated with protein kinase C, cyclin-dependent protein kinase (CDC2), and protein kinase A (PKA), respectively, and very less with the ataxia telangiectasia mutated (ATM). The most common site for phosphorylation was serine, in comparison with theorine and tyrosine (Table S2).

### 3.2. Phylogeny and Sequence Logos of GbNHX Genes with Different Species

**Figure 2:**
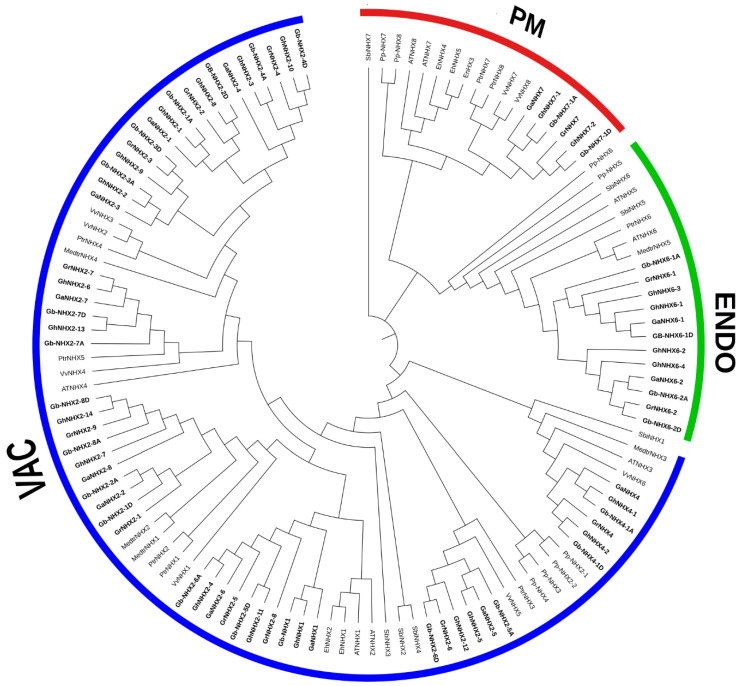
Phylogenetic tree of sodium transporters between the NHX transporters of 11 plant species by the neighbor-end joining method using MEGA 10.0. The tree divides all the 125 NHX genes into three groups based on their subcellular localization. Prefixes such as Gh, Gb, Gr, Ga, At, Vv, Ptr, Sb, Medtr, Eh, and Pp were used before the name of the species *G. hirsutum*, *G. barbadense*, *G. raimondii*, *G. arboreum*, *A. thaliana*, *V. vinifera, P. trichocarpa*, *S. bicolor*, *M. truncatula*, *E. halophilum*, and *P. patens*, respectively. *G. barbadense* genes are represented by bold letters. The amino acid sequences used in phylogenetic analysis are provided in Table S1.

In order to find the evolutionary relationship among the *NHX* genes, the protein sequences from 11 different plant species, including 4 gossypium species, *G. hirsutum*, *G. barbadence*, *G. arboreum*, and *G. raimondii*; 5 dicotyledonous angiosperms, *A. thaliana*, *V. vinifera*, *P. trichocarpa*, *M. truncatula*, and *E. halophilum*; one monocotyledonous angiosperm, *S. bicolor*; and one bryophyte, *P. patens*, were retrieved. A maximum likelihood tree was constructed among 123 *NHX* genes of the above-mentioned plant species. The phylogenetic tree depicted a direct relation with the subcellular localization, as all the *NHX* transporter proteins from different species clustered in three clades based upon their predicted location—viz., VAC (vacuolar membrane-bounded), ENDO (endomembrane-bounded), and PM (plasma membrane-bounded). Moreover, the VAC class has 85 genes, as most types of *NHX* genes (*NHX1*, *2*, *3*, and *4*) from different species are present on the vacuolar membrane, while ENDO has 20 and the PM class has 18 genes. Among the gossypium species, the VAC class has *NHX1*, *NHX2*, and *NHX4*; the ENDO class has *NHX6*; and the PM class has *NHX7* (Figure 2). To investigate the amino acid changes in the *NHX* domain across four cotton species, we generated the sequence logos of conserved amino acids. We found that many sequence logos were highly conserved across the N and C termini among different species. Within a species, the *NHX* domain of *G. raimondii* has the most conserved sequences (Figure S3).

### 3.3. Comparison of Motifs and Physical Genome Mapping of NHX Genes in G. barbadense and G. hirsutum

**Figure 3:**
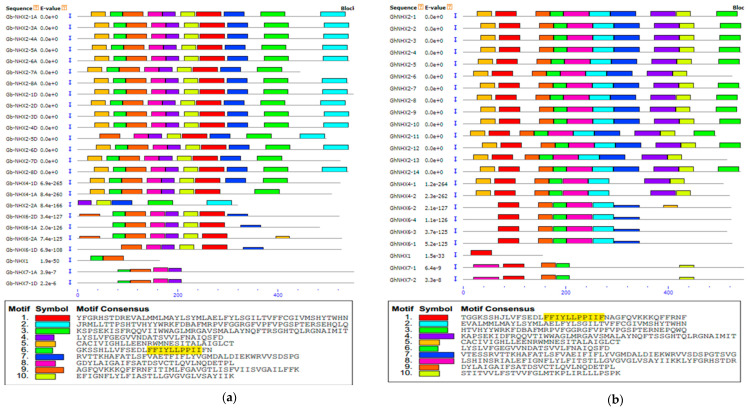
Conserved motif analysis of the *NHX* genes. (**a**) Motifs of the *GbNHX* Amiloride binding site) are represented by motif 7. (**b**) Motifs of the *GhNHX* Amiloride binding site are shown by motif 1. The sequence for amiloride binding site ((L/F)FF(I/L)(Y/F)LLPPI is highlighted.

A motif prediction carried out by MEME with 0–10 motif sites showed that all of the *G. barbadense NHX* transporters except one (*Gb-NHX2-2A*) have an amiloride binding motif, while in the case of *G. hirsutum*, all transporters have this motif (Figure 3a,b). To further investigate the presence of this motif in the *NHX* genes of other species, we aligned 99 amino acid sequences from the gossypium species, *V. vinifera*, *M. trunculata*, *A. thaliana*, and *P. trichocarpa*. Our results showed that almost all (97) the *NHX* transporters have an amiloride binding site, except *Gb-NHX2-2A* and *GaNHX6-1* of *G. barbadense* and *G. arboreum*, respectively (Figure S4). The physical mapping of the *NHX* transporters on the corresponding chromosomal loci in four Gossypium species showed that the *NHX* genes are scattered on both the A and D genomes. In *G. barbadense*, 12 genes were mapped on the At sub-genome, while 13 were mapped on the Dt sub-genome. In case of *G. hirsutum*, the At sub genome has 11 and the Dt sub-genome has 12 *NHX* genes. In both the allotertaploid species, A01, A09, A11, D01, D02, D09, and D11 have two, while A02, A03, A06, A12, A13, D06, D07, D12, and D13 have one *NHX* transporter each. Chromosomal mapping also showed some differences among both species, with only *G*. *barbadense* having one transporter on A08 and D08. Moreover, in *Gb* and *Gh* two *NHX* transporters were present on the chromosomes D01, DO9, and D11 each, while *G. raimondii*, the progenitor of the D genome, has no member on these chromosomes (Table 2, Figure S5).

### 3.4. Synteny Analysis and Ka/Ks Ratio of NHX in Cotton Species

To investigate the relationship among allotetraploid *G. barbadense* and its diploid ancestors *G. arboreum* and *G. raimondii*, a neighbor-end joining tree was constructed (Figure S6). The clusters formed in the tree with the same type of *NHX* genes from all three species provide evidence that *G. barbadense* is the result of hybridization between the two diploid cotton species, *G. arboreum* and *G. raimondii*.

**Figure 4:**
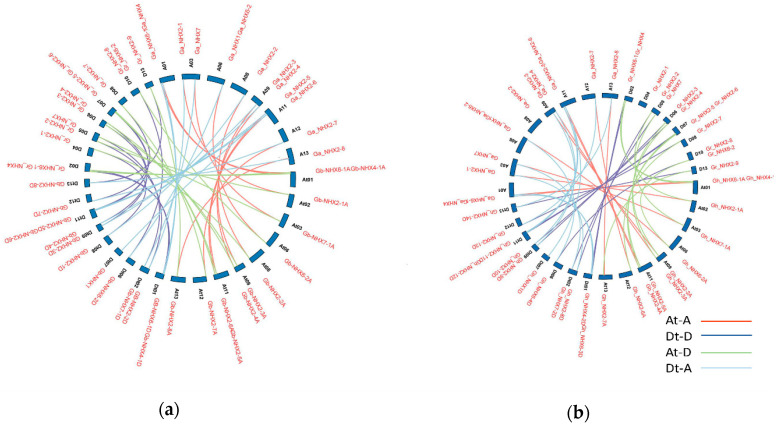
Collinearity analysis of *G. barbadense* (**a**) and *G. hirsutum* (**b**) (A and D) orthologs in the *G. arboreum* (A Chr) and *G. raimondii* (D Chr) genomes. Orthologs are connected by colored lines.

Being an allotetraploid, upland cotton is a model crop species to study natural polyploidy [78]. To study the relationship between the *GbNHX* and *GhNHX* genes, orthologous/paralogous genes pairs were identified for the At and Dt sub-genomes. In accordance with previous findings, our study also demonstrated that the At as well as the Dt sub-genomes have orthologs in the A (*G. arboreum*) or D (*G. raimondii*) genomes (Figure 4a,b). The synteny analysis showed a total of 30 gene duplication events in *G.barbadense*, while there were 31 in *G. hirsutum* on the basis of a whole-genome analysis (Table 3). Most of the *GbNHX* genes showed whole-genome or segmental duplication. Furthermore, to estimate the selection pressure on the Gossypium *NHX* transporters during the evolutionary time, we calculated the Ka and Ks values and Ka/Ks ratio in both tetraploid species. The Ka/Ks ratio for most of the genes was less than 1, while for only three (*Gb-NHX2-2A, Gb-NHX2-7D*, and *Gh_NHX6-3D*) was it more than 1 (Table S4). This indicates that the cotton *NHX* genes have been subjected to strong purifying selection. Interestingly, an expression analysis also revealed that *G. barbadense Gb-NHX2-7D* and *G. hirsutum Gh-NHX6-3D* have a higher expression in different tissues under salinity stress.

### 3.5. Promoter Analysis of G. barbadense and G. hirsutum NHX Genes

**Figure 5:**
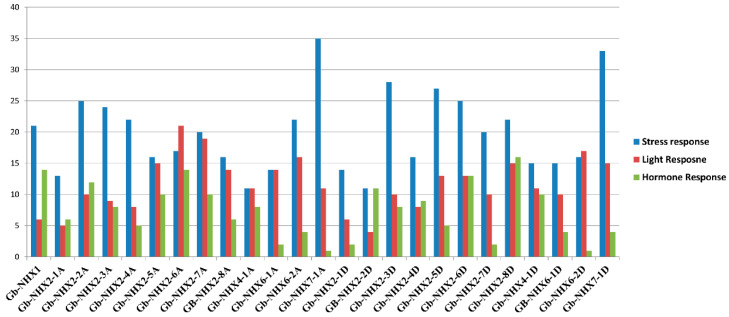
Cis-elements of the NHX transporters. Vertical axis represents the number of Cis-elements, and the horizontal axis shows the genes name.

Cis-acting elements in the promoter region play a key role in defining the plant response towards stress and light and in growth regulation. To investigate the transcriptional potential of the Na^+^/H^+^ transporter genes, we analyzed and predicted the Cis-elements in 2000 bp promoter regions upstream of the start codon. Besides the abundant amount of core promoter/enhancer elements—i.e., CAAT-Box (CAAT, CAAAT, and TGCCAAC) and TATA-box (ATTATA, TAAAGATT, TATTTAAA, TATA, ccTATAAAaa, TATACA), with a total number of 806 and 1178, respectively—we found different elements related to stress, light, and hormone response. Interestingly, the *NHX* genes contained a larger number of Cis-elements related to stress response than to light and hormone response, indicating their role in stress regulation. The water and drought response elements MYB (CAACCA/TAAC/TAACTG) and MYC (CAATTG/TCTCTTA/TCTCTTA) were the most abundant among all the elements present, with a total number of 89 (12%) and 72 (10%), respectively (Table S5). In *G. barbadense*, 21 *GbNHX*s have AREs (anaerobic-responsive elements); 17 have STREs (stress-responsive elements); 10 contained the WUN-motif (wound-response element); and 9 *GbNHX*s had a W-box, which is involved in pathogen response [79]. Meanwhile, the *G. hirsutum* NHXs have comparatively less putative Cis-elements, with 17 *GhNHX*s having AREs and 15 having STREs, while the WUN-motif and W-box were found in 9 and 7 *GhNHX*s, respectively. The promoter region of *Gb-NHX7-1A* (*Gbar_A03G012870*) and *Gb-NHX7-1D* (*Gbar_D02G014810*) has a maximum number of stress-responsive Cis-elements (Figure 5).

### 3.6. Expression Pattern of G. barbadense NHX Genes and Its Comparison with G. hirsutum under Salt Stress

**Figure 6:**
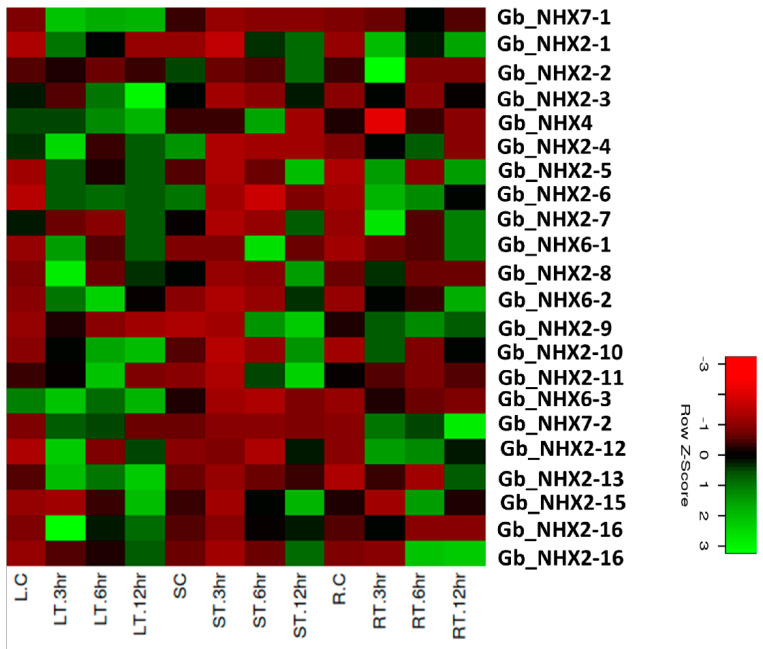
Relative expression level analysis of NHX in Gossypium Hirsutum. Relative expression of different NHXs is shown under the controlled conditions and salinity stress in different tissues at different time intervals. *Y*-axis shows the gene names and *X*-axis represents the tissue and time interval. Colors represent the expression level normalized against the control tissues. LC: leaf control; RC: root control; SC: stem Control; LT: treated leaf; RT: treated root; ST: treated stem.

**Figure 7:**
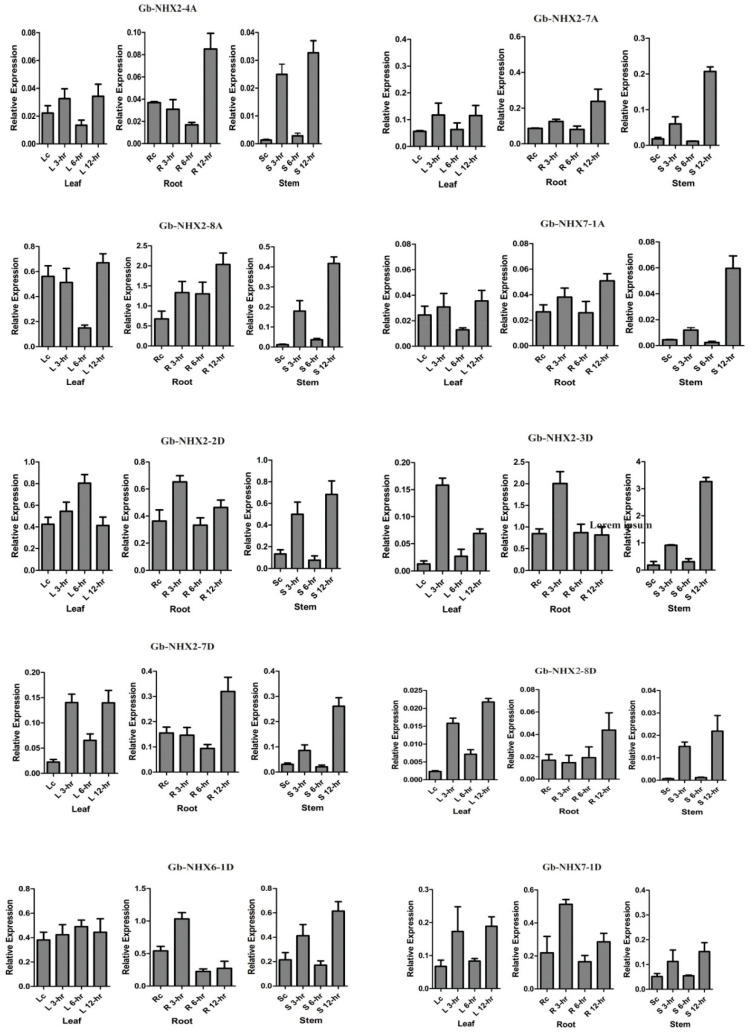
Relative expressions of 10 GbNHX genes under salinity stress based on a qRT-PCR. The values are the means ± standard deviations (SD) of three replicates. Gene specific primers list is provided in Table S3.

The expression pattern of *NHX* genes under salinity stress was checked to investigate their potential role in *G. barbadense* and was compared to that of *G. hirsutum*. Previously, *G. barbadense* was found to be more tolerant to salinity than *G. hirsutum* [80,81]; studies showed that it has more lateral roots under a stress environment [82]. We used qRT-PCR for the expression analysis of all the *NHX* transporters in *G.barbadense* and *G. hirsutum* in the root, stem, and leaf tissue at 0, 3, 6, and 12 h time intervals. Our results revealed that in case of *G. barbadesne*, most genes show a higher expression level in the stem tissue, while in *G. hirsutum*, more genes are expressed in the roots and leaves, with a less significant expression in the stem under stress as compared with the control (Figure 6 and Figure 7). Ten *GbNHX* genes—*Gb-NHX2-4A*, *Gb-NHX2-7A Gb-NHX2-8A*, *Gb-NHX7-1A*, *Gb-NHX2-2D*, *Gb-NHX2-3D*, *Gb-NHX2-7D*, *Gb-NHX2-8D Gb-NHX6-1D* and *Gb-NHX7-1D*—with a higher expression were further analyzed (Figure 7). The genes showed differential expressions in different tissues. Almost all the genes showed a maximum expression at 12h in different tissues. Our results also showed that *Gb-NHX2-7A*, *Gb-NHX2-3D*, and *Gb-NHX2-7D* have a higher number of stress-related Cis-elements in their promoter region that could be related to high expression under stress. Additionally, the Ka/ks ratio revealed that *Gb-NHX2-7D* underwent positive selection. Moreover, we observed that the *NHX2* homologues in both species have a high expression under salinity stress at higher time intervals, followed by *NHX7*. In *G. barbadense*, the plasma membrane-bounded *NHX7* has a high expression level in all tissues under stress.

### 3.7. Protein-Protein Interaction Prediction and GO of GbNHX Genes

**Figure 8:**
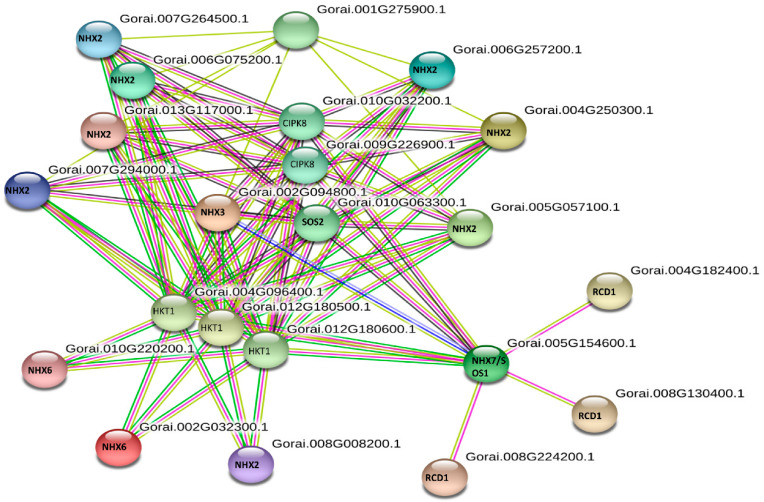
String analysis of *GbNHX* interacting proteins.

**Figure 9:**
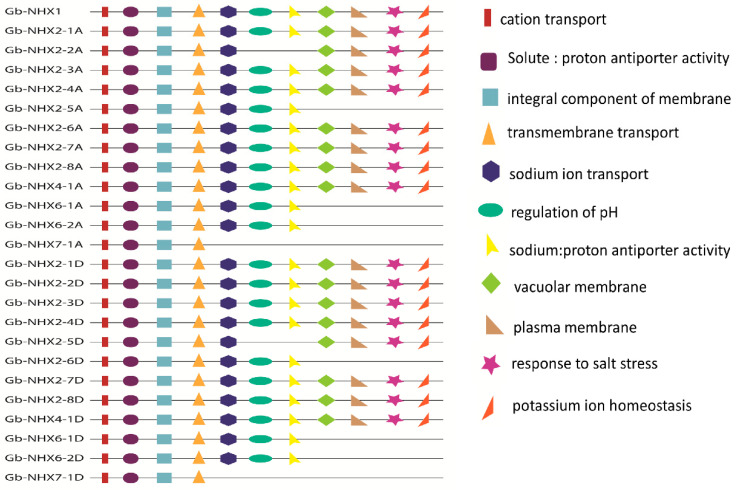
Gene ontology (GO) terms of GbNHX genes. Different GO terms are represented by different shapes.

On the string database, only the *Gossypium raimondii* (Gr) protein-protein interaction network was available until now. Thus, in this study we used the homolog gene between *GrNHX* and *GbNHX* to search in the database. The *GrNHX* homolog gene and interacted protein were used to construct a network to predict the *GbNHX* protein-protein interaction network. We observed that the *Gossypium NHX* proteins interact with other proteins, such as *HKT1*, conferring salinity tolerance and RCD 1 (Radical-Induced Cell Death protein 1), which supports chloroplasts against high ROS (Reactive oxygen species). The *NHX* protein also interacted with calcineruin B-like proteins (CBL10) and some CBL-interacting protein kinases (CIPKs), such as *CIPK8* and *CIPK 24*. Meanwhile, *NHX7/SOS1* and SOS2, interacting with almost all proteins, were found to be the centers of interaction (Figure 8). When single proteins were subjected to analysis individually, they showed a similar kind of interaction with related proteins involved in stress tolerance (Figure S7). Moreover, the gene ontology (GO) of the *GbNHX* gene showed that they are enriched in 11 GO terms related to potassium ion homeostasis, the response to salt; the regulation of pH; sodium: proton antiporter activity; solute: proton antiporter activity; cation transport; transmembrane transport; the integral component of membrane; sodium ion transport; the vacuolar membrane; and the plasma membrane (Figure 9, Figure S8, Table S6).

## 4. Discussion

Salinity causes ion toxicity and physiological drought, thus limiting the growth and productivity of plants [2]. Recently, the availability of high-quality de novo genome assemblies for *G. arboreum* [56] and allotetraploids cottons [83] generate new opportunities for precise genome-wide studies in cotton. The *NHX*s genes present in plant cells maintain the ionic homeostasis by playing their role in the extrusion of Na+ ions out of the cell and the compartmentalization of Na^+^ ions into the vacuole [84]. In the current study, a total of 25 with different types—i.e., *NHX1, NHX2, NHX4, NHX6*, and *NHX7*—of sodium transporters have been identified in *G. barbadense*, based on the Na^+^_H^+^_Exchanger domain (Table 1).

A bioinformatics analysis showed that the *NHX* members in *G. barbadense* can be divided into three categories depending upon their subcellular location, with *NHX7* localized in the plasma membrane, *NHX6* in the endomembrane, and the others in the tonoplast. In Arabidopsis, both *NHX7* and *NHX8* are localized in the plasma membrane [85], while *NHX5* and *NHX6* are present in the endomembrane [29]. However, no *NHX5* and *NHX8* were observed in the Gossypium species in this study (Figure 2). Subcellular localization could be a key factor in defining the function of *NXH* transporters. *NHX* members located on both the plasma membrane and tonoplast play their role in the exclusion and compartmentalization of excess Na+ and maintain ionic homeostasis. Moreover, some *NHX* members that are endomembrane-bounded were found to be vital for cellular cargo trafficking, growth development, and the regulation of protein processing [13,29]. The phylogenetic analysis indicated that *GbNHX* has paralogous or orthologous groups with other Gossypium species members. The *NHX* genes in *P. trichocarpa* [69], *S. bicolor* [67], and *B. vulgaris* [86] showed three phylogenetic clusters based on their location in the cell; we found the same results for cotton *NHX* transporters. An amiloride binding site (L/F)FF(I/L)(Y/F)LLPPI, a typical feature of *NHX* transporters in plants [87,88], is present in the N-terminal of these proteins; the presence of amiloride even in a micro amount in the Na^+^/H^+^ exchangers inhibits the transport of Na+ transport [89]. This site was found in most of *G. barbadense* transporters, such as Arabidopsis and poplar [69] (Figure S2).

During the cotton evolution period, the occurrence of a gene duplication event led to the creation of new genes [90]. The origin of multi-gene families has been attributed to a region-specific gene duplication that occurred in upland cotton [53]. The presence of two or more genes on the same chromosome reveal the possibility of a tandem duplication event, while the genes present on different chromosomes result in a segmental duplication event. The duplication of genes increase the functional divergence, which is an essential factor in adoptability under changing environmental conditions [91]. The Ka/Ks ratio is a measure used to examine the mechanisms of gene duplication evolution after divergence from their ancestors. The Ka/Ks ratio gives an insight into the selection pressure on amino acid substitutions, with a Ka/Ks ratio < 1 indicating a purifying selection, while a ratio > 1 suggests the possibility of positive selection. Wang et al. [92] showed in *T. aestivum* and *TaBT1* that the positive selection of a gene during evolution increases its potential and has more transcription levels under stress conditions. Almost all except 3 out of 31 duplication events occurred in the *G.barbadense* and *G. hirsutum* NHX transporters showing a <1 substitution value, indicating that these genes underwent a positive Darwinism or purifying selection [93] (Table 3).

The promoter region of *G. barbadense* and *G. hirsutum NHX* transporters has light, stress, and hormone- and development-responsive Cis-acting elements, showing that these genes are not only regulated by abiotic stress but also by different hormones (Table S5). However, the number of stress-responsive Cis-elements exceeds the others, indicating their major role in abiotic stress response (Figure 5). Similar to Arabidopsis [67], abscisic acid-responsive elements (ABRE), auxin-responsive elements, fungal-responsive elements, circadian elements, low temperature-responsive elements (LTR), heat shock elements (HSE), and MYB Cis-elements were noticed in the *Gossypium barbadesne NHX* gene promoter. The β-glucoronidase gene driven by the AtMYB2 promoter in Arabidopsis was found to be inducible by osmotic stresses [94]. G-box elements that act as positive regulators of early leaf senescence in rice [95] were also detected in the promoter regions of Gossypium *NHX* transporters, implying that these genes also modulate the leaf senescence.

In plants, sodium-proton antiporters facilitate both Na^+^/H^+^ and K^+^/H^+^ exchanges, therefore contributing to both stress tolerance and K^+^ nutrition [25,26,96]. *NHX* genes have been reported to enhance salinity tolerance in different species, such as *A. thaliana* [37], *B. vulgaris* [97], *S, lycopersicum* [40,98], *H. vulgare* [99], *Z. maize* [100], *T. aestivum* [101], *G. max* [102], *O. sativa* [103,104], and *S. bicolor* [67]. Our study revealed that in *G. barbadense* and *G. hirsutum*, the *NHX* genes express differentially in different tissues at different time intervals under salinity stress. Ma et al. [105] also observed different expression levels of *NHX* genes in different tissues of *V. vinifera* L. The vac-class *NHX2* homologues in cotton show a higher expression under salinity stress. When *R. trigyna* is exposed to salinity stress, an increase in the transcription level of the vac-class *RtNHX1* gene in leaves was observed [106]. A similar kind of expression pattern was observed in sweet potato, *IBNHX2* [107] and in *T. aestivum, TaNHX3* [108] under the salt treatment.

The plasma membrane-bounded *NHX7/SOS1* gene helps in the exclusion of Na^+^ ions from the cell to regulate ionic homeostasis [5,109]; it was validated in the present study that *Gb-NHX7* showed a higher expression under the salinity stress. It is noticeable that its expression is higher in roots at all time periods than in other tissues. Similar results have been noticed in *Salicornia brachiate* [110], *P. tenuiflora* [111], and *Z. xanthoxylum* [112], where plasma membrane-bounded NHX7/SOS1 showed a higher expression in roots than in shoots and was further increased at a higher salt stress. These results proposed that *GbNHX7* could be responsible for the long distance transport of Na^+^ ions, but the detailed mechanism is still to be explored.

The protein-protein interaction showed that *GbNHX* interacted with many other proteins. The tails on the C-terminal of *SOS1* and *NHX1* were revealed to be essential for protein-protein interaction by Quintero at el. [113]. In Arabidopsis, *SOS1* interacts with *RCD*1 (radical-induced cell death protein 1) to increase the tolerance against oxidative stress caused by ROS [114]. Our hypothesis also indicated the presence of interaction between *NHX7/SOS1* and *RCD1* to improve the salt tolerance ability of the plants. Moreover, in the present study *HKT1* was found to interact with almost all the *GbNHX* genes. Zhang et al. [111] observed that under considerably high salt concentrations when vacuoles have no more capacity to sequester Na^+^ ions, the *HKT1;5* is strongly expressed to increase the salinity tolerance by unloading excess Na^+^ ions from the xylem. The interaction between the CBL proteins and CIPK is also known to be involved in enhancing the ability of the plant to withstand salt stress [115]. Kim et al. [116] observed that *CIPK24/SOS2* make a complex with *CBL3/SOS3* that phosphorylates the *NHX7/SOS1* localized in the plasma membrane to pump Na^+^ ions out from the cell. The single protein-protein interaction is this study also infers a similar kind of results, showing the interaction of *NHX7/SOS1* with *CIPK24* and *CIPK8*, besides others.

## 5. Conclusions

A genome-wide study of *G. Barbadense* revealed the presence of four types (*NHX2*, *NHX4*, *NHX6*, and *NHX7*) of sodium transporters that can be categorized as plasma membrane (*GbNHX7*), endomembrane (*GbNHX6*), and vacuolar (*GbNHX1, 2*, and *4*), based on their location. The amiloride-binding site (FFIYLLPPI) is found in all *GbNHX* genes. The high number of stress related Cis-acting elements observed in promoters show their role in tolerance against abiotic stresses. A chromosomal localization and collinearity analysis showed the purified selection and evolution of gossypium *NHX* genes. An in silico PPI network analysis showed that only *GbNHX7* interacts with CBLs and CIPKs, suggesting this protein might be the primary *NHX* involved in the CBL-CIPK pathway during the salt stress response. The gene ontology (GO) showed that these genes are involved in the proton antiport, sodium ion transport across the membrane, and salinity response activities. A tissue-specific qRT-PCR-based expression analysis of *NHX* antiporters revealed that they are more expressed under stress conditions in comparison with control conditions. The expression pattern was also different in different tissues of *G. barbadense* and *G. hirsutum*. The higher expression of vac-class in leaves may also be responsible for the deposition of salts, especially in older leaves. These results showed that these genes could be involved in various developmental processes and stress responses by maintaining the turgor pressure, pH, and ionic homeostasis. Our findings would be useful in selecting candidate genes for functional validation in relation to high soil salinity stress tolerance for the improvement of crop plants.

## Figures and Tables

**Table 1:** Characteristics of the *G. barbadense* sodium-proton antiporter (NHX) transporters.

Gene Name	Gene ID	Protein (aa)	CDS (bp)	MW (kDa)	pI	Localization	Na+/H+ Exchanger Domain (Start–End)
Gb-NHX1	Gbar_D07G012760	164	1455	18.666	10.438	Vac	Nil
Gb-NHX2-1A	Gbar_A02G004630	535	1527	59.063	8.663	Vac	22–437
Gb-NHX2-2A	Gbar_A08G023430	320	1608	35.548	10.181	Vac	7–212
Gb-NHX2-3A	Gbar_A09G007060	542	3459	59.844	7.858	Vac	29–444
Gb-NHX2-4A	Gbar_A09G025000	541	1587	59.721	9.05	Vac	25–445
Gb-NHX2-5A	Gbar_A11G028010	540	963	59.586	6.989	Vac	16–440
Gb-NHX2-6A	Gbar_A11G025170	543	1629	60.106	7.657	Vac	24–445
Gb-NHX2-7A	Gbar_A12G000720	445	1626	49.371	8.063	Vac	17–420
Gb-NHX2-8A	Gbar_A13G011300	541	1632	59.706	8.552	Vac	25–444
Gb-NHX4-1A	Gbar_A01G007690	508	1623	56.977	7.591	Vac	16–426
Gb-NHX6-1A	Gbar_A01G002880	484	1338	53.246	6.795	Endo	25–433
Gb-NHX6-2A	Gbar_A06G019530	528	1626	58.41	5.514	Vac	25–437
Gb-NHX7-1A	Gbar_A03G012870	1152	1584	128.07	6.878	PM	29–445
Gb-NHX2-1D	Gbar_D08G024100	551	1578	61.315	8.457	Vac	30–434
GB-NHX2-2D	Gbar_D02G005160	535	1608	59.178	8.453	Vac	22–437
Gb-NHX2-3D	Gbar_D09G006790	542	3459	60.015	8.731	Vac	29–444
Gb-NHX2-4D	Gbar_D09G024630	541	1572	59.705	9.175	Vac	29–444
Gb-NHX2-5D	Gbar_D11G026100	497	495	55.119	7.009	Vac	4–406
Gb-NHX2-6D	Gbar_D11G028500	542	1656	59.758	6.42	Vac	19–448
Gb-NHX2-7D	Gbar_D12G000860	525	1629	58.126	8.549	Vac	25–444
Gb-NHX2-8D	Gbar_D13G011070	541	1626	59.715	8.554	Vac	31–442
Gb-NHX4-1D	Gbar_D01G007950	525	1494	59.139	7.62	Vac	21–441
GB-NHX6-1D	Gbar_D01G003050	527	1629	58.056	5.978	Endo	28–437
Gb-NHX6-2D	Gbar_D06G020390	523	1578	57.72	5.494	Endo	28–432
Gb-NHX7-1D	Gbar_D02G014810	1152	1626	128.14	6.764	PM	31–443

aa: amino acid; pI: isoelectric point; MW: molecular weight; Vac: vacuole; Pm: plasma membrane; Endo: endomembrane.

**Table 2:** Chromosomal location of the *NHX* genes in the Gossypium species.

Chromosome	*G. arboreum*	*G. raimondii*	*G. barbadense*	*G. hirsutum*
A01	Ga_NHX6-1		Gb-NHX6-1A	Gh_NHX6-1A
A01	Ga_NHX4		Gb-NHX4-1A	Gh_NHX4-1A
A02			Gb-NHX2-1A	Gh_NHX2-1A
A03	Ga_NHX2-1			
A03	Ga_NHX7		Gb-NHX7-1A	Gh_NHX7-1A
A06	Ga_NHX1			
A06	Ga_NHX6-2		Gb-NHX6-2A	Gh_NHX6-2A
A08	Ga_NHX2-2		Gb-NHX2-2A	
A09	Ga_NHX2-3		Gb-NHX2-3A	Gh_NHX2-2A
A09	Ga_NHX2-4		Gb-NHX2-4A	Gh_NHX2-3A
A11	Ga_NHX2-5		Gb-NHX2-6A	Gh_NHX2-4A
A11	Ga_NHX2-6		Gb-NHX2-5A	Gh_NHX2-5A
A12	Ga_NHX2-7		Gb-NHX2-7A	Gh_NHX2-6A
A13	Ga_NHX2-8		Gb-NHX2-8A	Gh_NHX2-7A
D01			Gb-NHX6-1D	Gh_NHX6-1D
D01			Gb-NHX4-1D	Gh_NHX4-1D
D02		Gr_NHX6-1	Gb-NHX2-2D	Gh_NHX2-1D
D02		Gr_NHX4	Gb-NHX7-1D	Gh_NHX7-1D
D04		Gr_NHX2-1		
D05		Gr_NHX2-2		
D05		Gr_NHX7		
D06		Gr_NHX2-3	Gb-NHX6-2D	Gh_NHX6-2D
D06		Gr_NHX2-4		
D07		Gr_NHX2-5	Gb-NHX1	Gh_NHX1
D07		Gr_NHX2-6		
D08		Gr_NHX2-7	Gb-NHX2-1D	
D09			Gb-NHX2-3D	Gh_NHX2-2D
D09			Gb-NHX2-4D	Gh_NHX2-3D
D10		Gr_NHX2-8		
D10		Gr_NHX6-2		
D11			Gb-NHX2-5D	Gh_NHX2-4D
D11			Gb-NHX2-6D	Gh_NHX2-5D
D12			Gb-NHX2-7D	Gh_NHX2-6D
D13		Gr_NHX2-9	Gb-NHX2-8D	Gh_NHX2-7D

**Table 3:** Orthologous and paralogous gene pairs for *Gb* and *Gh.*

G. barbadense Orthologous/Paralogous		G. hirsutum Orthologous/Paralogous	
Gene ID	Gene ID	Gene ID	Gene ID
Gb-NHX6-1A	GB-NHX6-1D	Gh_NHX6-1	Gh_NHX2-2
Gb-NHX4-1A	Gb-NHX4-1D	Gh_NHX6-1	Gh_NHX2-3
Gb-NHX2-1A	Gb-NHX2-4A	Gh_NHX6-1	Gh_NHX6-3
Gb-NHX2-1A	Gb-NHX2-3A	Gh_NHX6-1	Gh_NHX2-9
Gb-NHX2-1A	GB-NHX2-2D	Gh_NHX6-1	Gh_NHX2-10
Gb-NHX2-1A	Gb-NHX2-4D	Gh_NHX4-1	Gh_NHX4-2
Gb-NHX2-1A	Gb-NHX2-3D	Gh_NHX2-1	Gh_NHX2-2
Gb-NHX7-1A	Gb-NHX7-1D	Gh_NHX2-1	Gh_NHX2-3
Gb-NHX6-2A	Gb-NHX6-2D	Gh_NHX2-1	Gh_NHX2-6
Gb-NHX2-2A	Gb-NHX2-6A	Gh_NHX2-1	Gh_NHX2-8
Gb-NHX2-2A	Gb-NHX2-1D.1	Gh_NHX2-1	Gh_NHX2-10
Gb-NHX2-2A	Gb-NHX2-8D.1	Gh_NHX2-1	Gh_NHX2-9
Gb-NHX2-4A	GB-NHX2-2D	Gh_NHX7-1	Gh_NHX7-2
Gb-NHX2-3A	GB-NHX2-2D	Gh_NHX2-2	Gh_NHX2-3
Gb-NHX2-4A	Gb-NHX2-4D	Gh_NHX2-2	Gh_NHX2-8
Gb-NHX2-3A	Gb-NHX2-3D	Gh_NHX2-2	Gh_NHX6-4
Gb-NHX2-6A	Gb-NHX2-8A	Gh_NHX2-2	Gh_NHX2-9
Gb-NHX2-6A	Gb-NHX2-1D	Gh_NHX2-3	Gh_NHX2-8
Gb-NHX2-6A	Gb-NHX2-5D	Gh_NHX2-3	Gh_NHX2-10
Gb-NHX2-5A	Gb-NHX2-6D	Gh_NHX2-3	Gh_NHX2-9
Gb-NHX2-6A	Gb-NHX2-8D	Gh_NHX2-4	Gh_NHX2-7
Gb-NHX2-7A	Gb-NHX2-7D	Gh_NHX2-4	Gh_NHX2-11
Gb-NHX2-8A	Gb-NHX2-1D	Gh_NHX2-4	Gh_NHX2-14
Gb-NHX2-8A	Gb-NHX2-5D	Gh_NHX2-5	Gh_NHX2-12
Gb-NHX2-8A	Gb-NHX2-8D	Gh_NHX2-6	Gh_NHX2-8
GB-NHX2-2D	Gb-NHX2-4D	Gh_NHX2-7	Gh_NHX2-11
GB-NHX2-2D	Gb-NHX2-3D	Gh_NHX2-7	Gh_NHX2-14
Gb-NHX2-1D	Gb-NHX2-5D	Gh_NHX2-8	Gh_NHX2-10
Gb-NHX2-1D	Gb-NHX2-8D	Gh_NHX2-8	Gh_NHX2-9
Gb-NHX2-5D	Gb-NHX2-8D	Gh_NHX6-4	Gh_NHX2-9
		Gh_NHX2-11	Gh_NHX2-14

## Supplementary Materials

The following are available online at https://www.mdpi.com/2073-4425/11/7/803/s1: Table S1: Phylogeny Sequences. Table S2: Phosphorylation sites and gene rename file. Table S3. qRT-PCR primer sequences. Table S4: Ka/Ks ratio of *Gb* and *Gh NHX* genes. Table S5: Cis-acting elements for *GBNHX* and *GhNHX*. Table S6: Gene Ontologies term. Figure S1: Transmembrane Domains for *GBNHX* proteins. Figure S2: Gene Structure for *GbNHX* and *GhNHX*. Figure S3: Sequence logos of four cotton species. Figure S4: Amiloride binding site in *NHX* genes. Figure S5: Chromosomal map four for Gossypium species. Figure S6: *Gb*, *Ga* and *Gr NHX* genes phylogenetic tree. Figure S7: Single protein-protein Interaction. Figure S8: GO terms graph.
