# Supplementary figures and images for: Genome-Wide Characterization and Expression Analysis of NHX Gene Family under Salinity Stress in Gossypium barbadense and Its Comparison with Gossypium hirsutum
Source: Genes (Basel). 2020 Jul 16;11(7):803. doi: 10.3390/genes11070803 (PMC7397021; doi:10.3390/genes11070803)

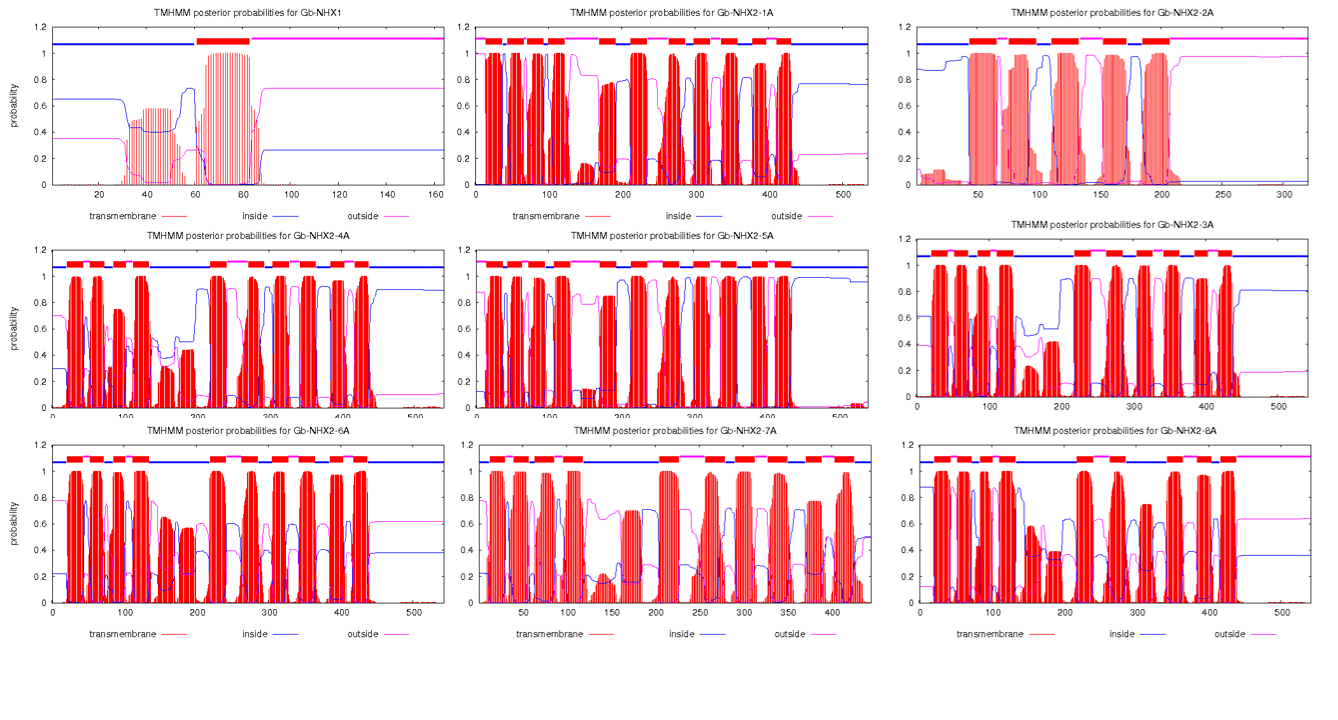

Supplement: Supplementary file 1 [file genes-11-00803-s001.zip › genes-850571 suppl/Figure S1a.tif]

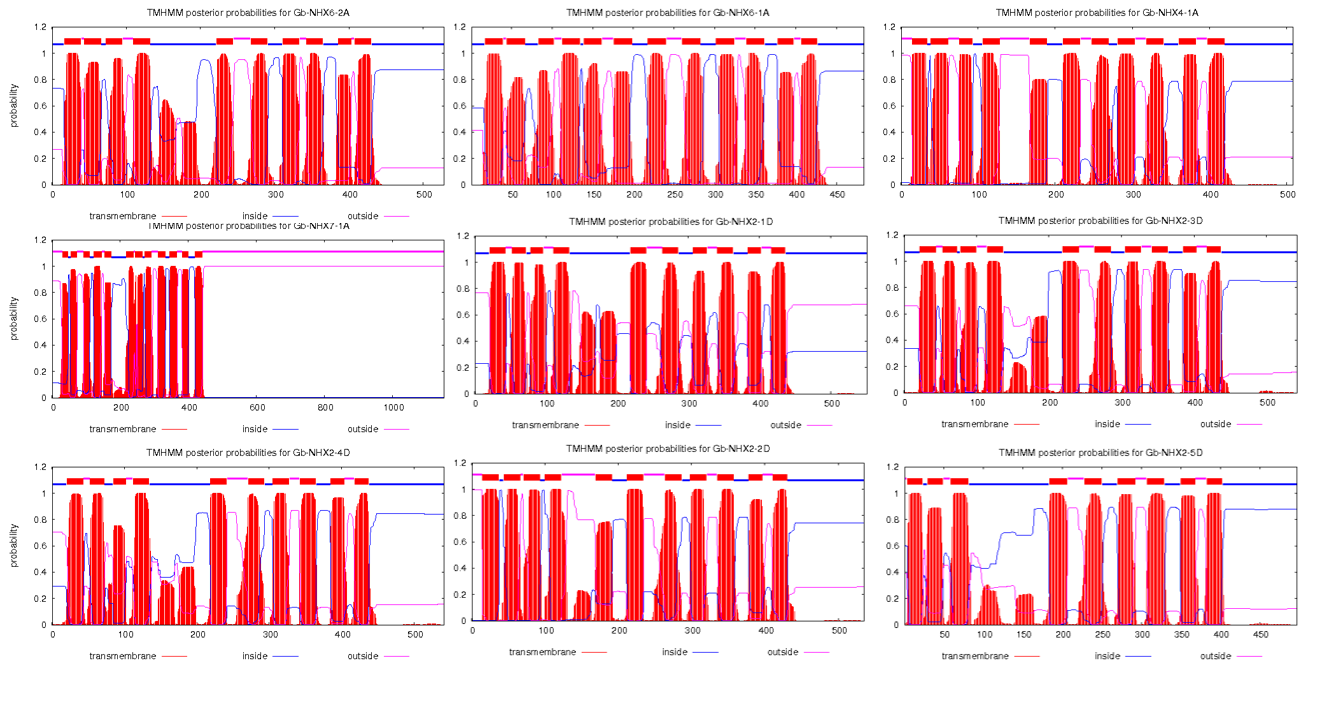

Supplement: Supplementary file 1 [file genes-11-00803-s001.zip › genes-850571 suppl/Figure S1b.tif]

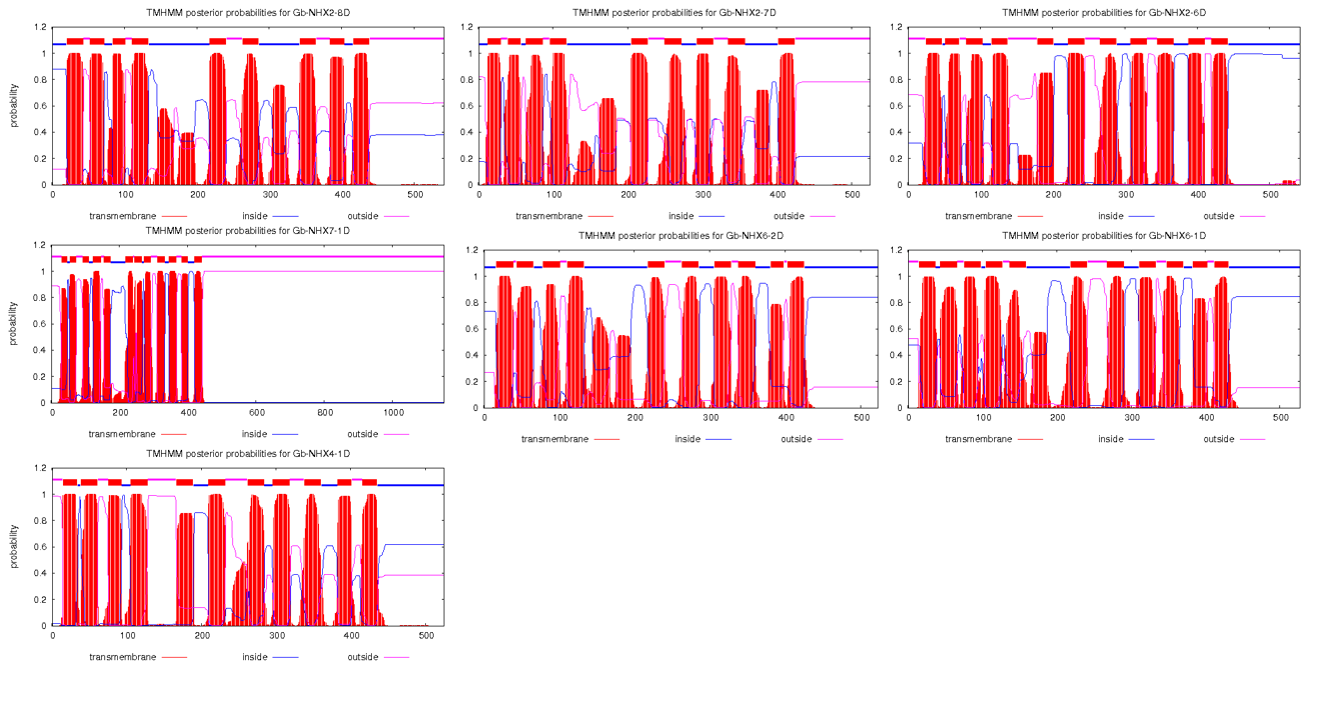

Supplement: Supplementary file 1 [file genes-11-00803-s001.zip › genes-850571 suppl/Figure S1c.tif]

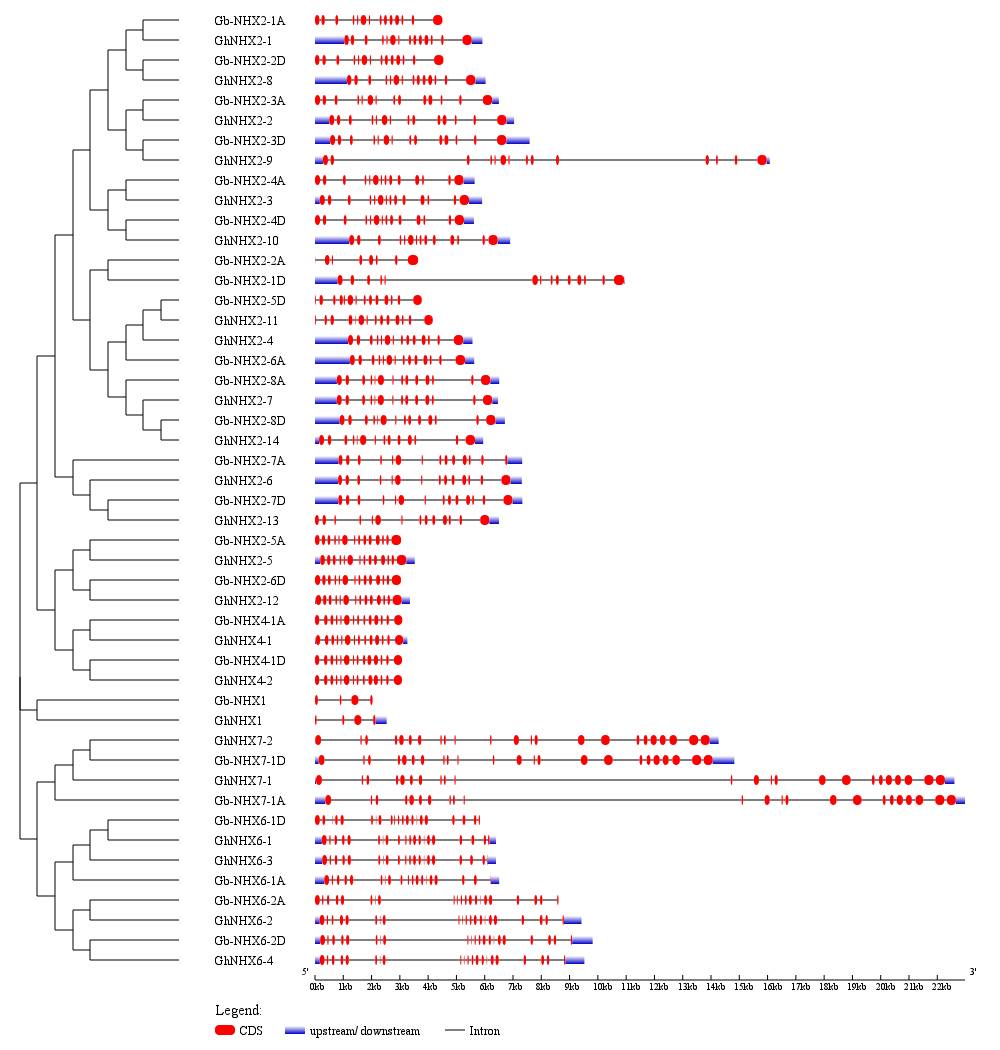

Supplement: Supplementary file 1 [file genes-11-00803-s001.zip › genes-850571 suppl/Figure S2 Gene structure Gb Gh.png]

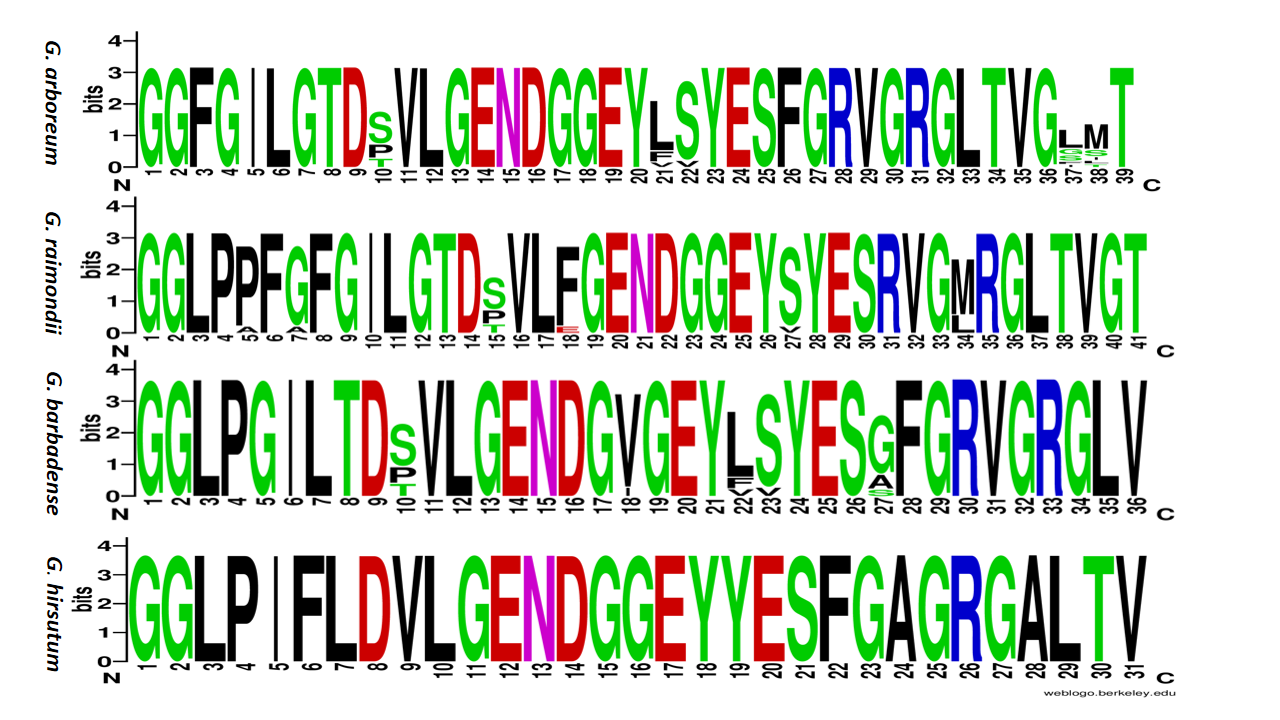

Supplement: Supplementary file 1 [file genes-11-00803-s001.zip › genes-850571 suppl/Figure S3 Sequence logos.png]

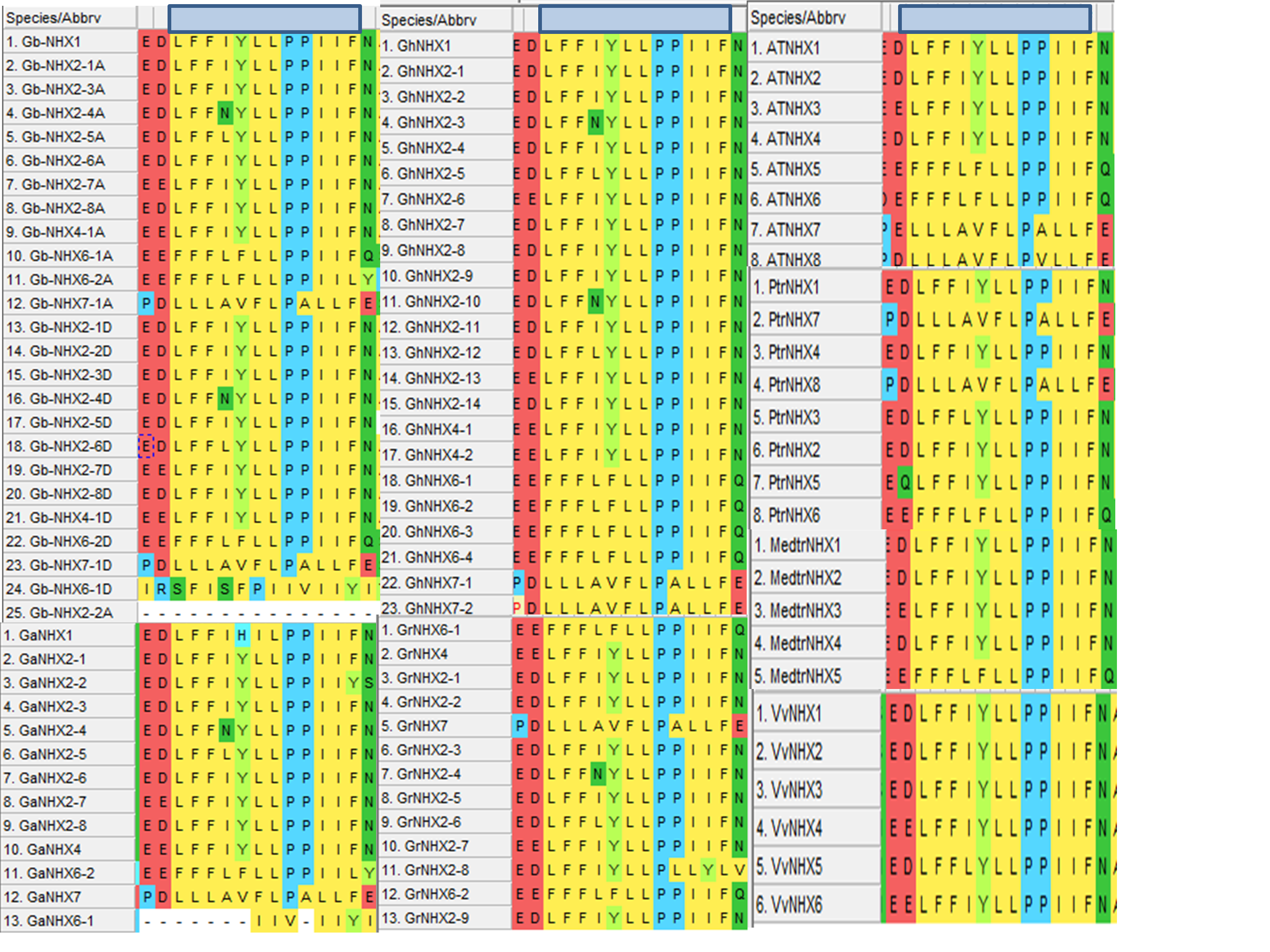

Supplement: Supplementary file 1 [file genes-11-00803-s001.zip › genes-850571 suppl/Figure S4 Amiloride binding Site.png]

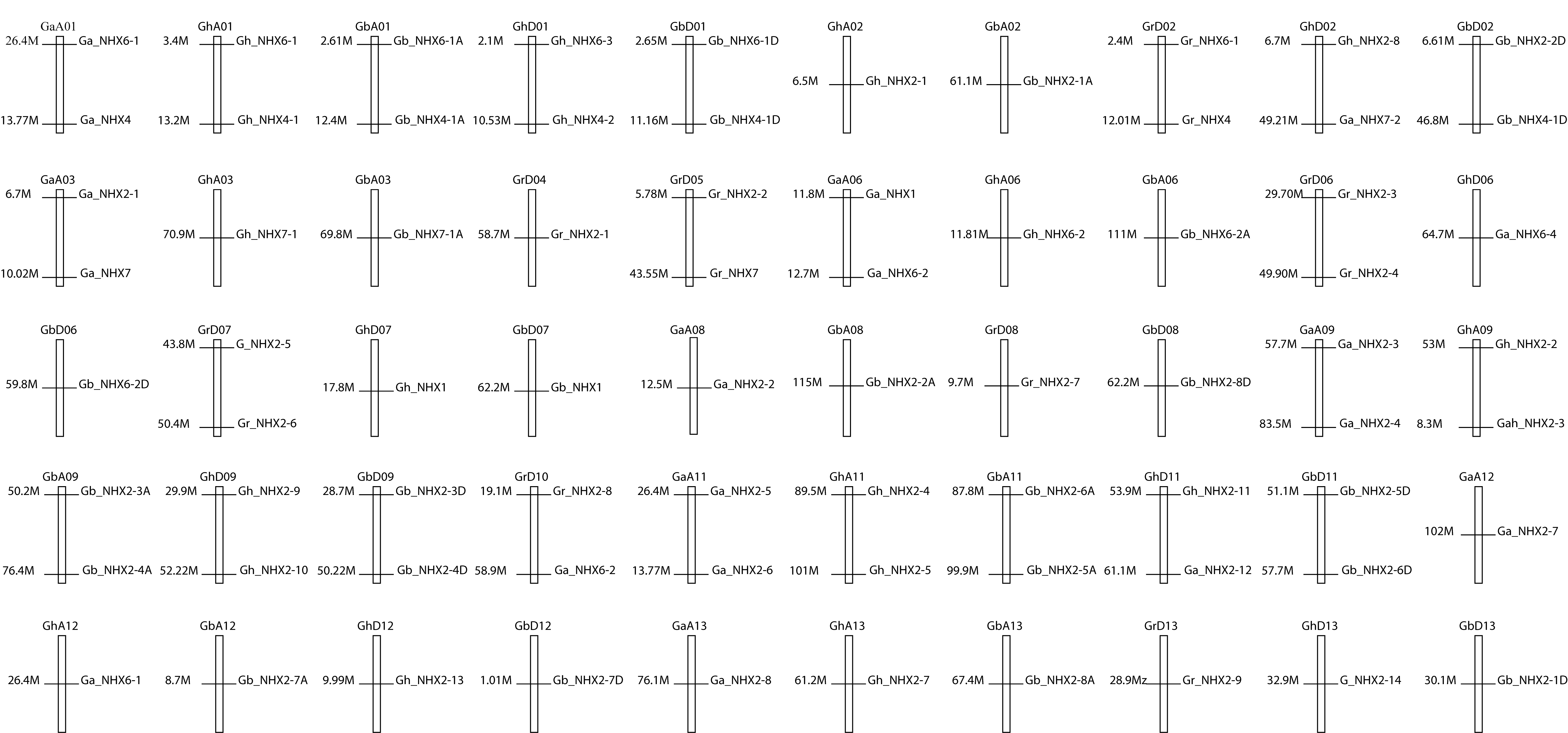

Supplement: Supplementary file 1 [file genes-11-00803-s001.zip › genes-850571 suppl/Figure S5 Chromosomal Map.tif]

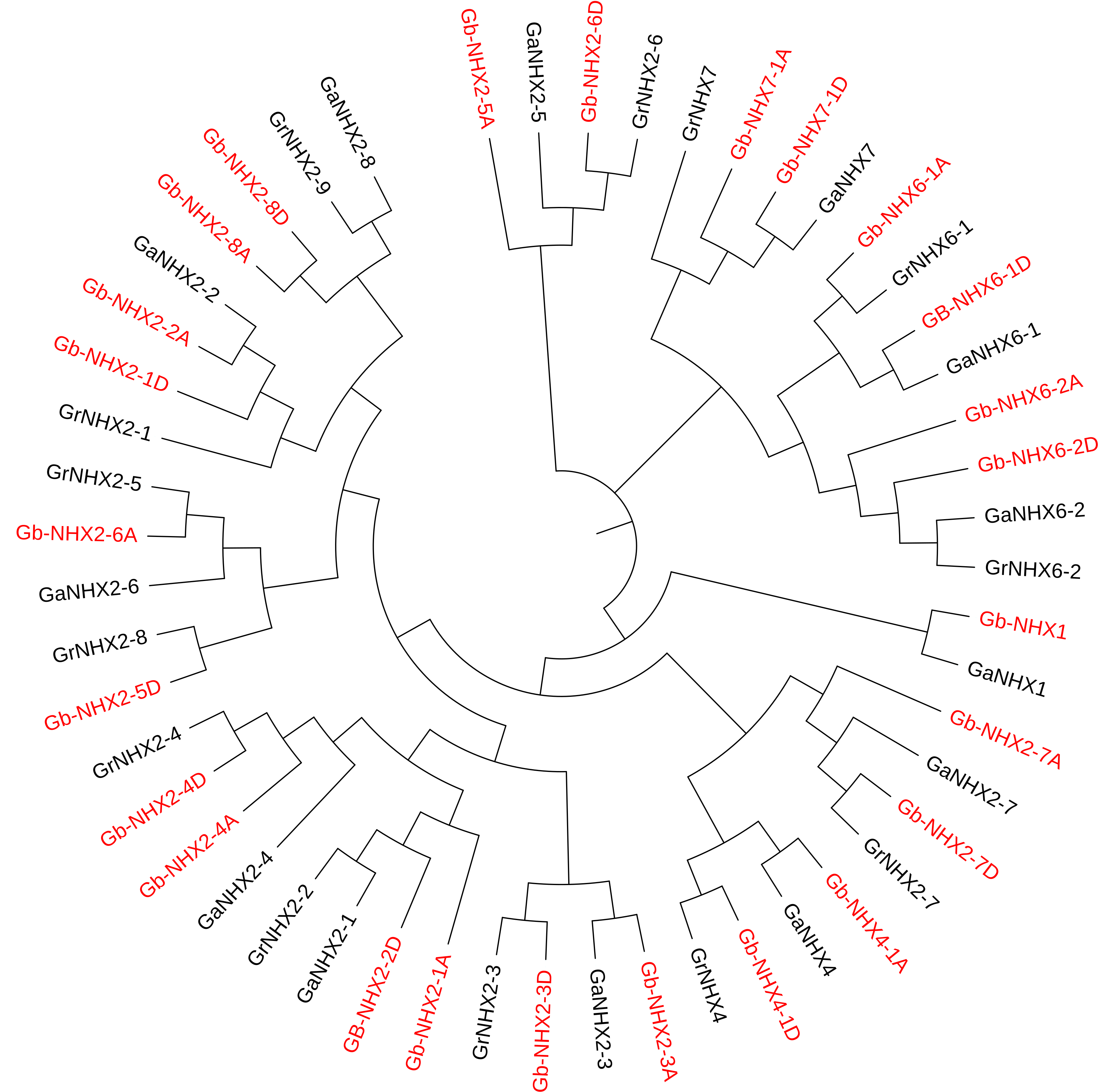

Supplement: Supplementary file 1 [file genes-11-00803-s001.zip › genes-850571 suppl/Figure S6 Gb Ga Gr Tree.png]

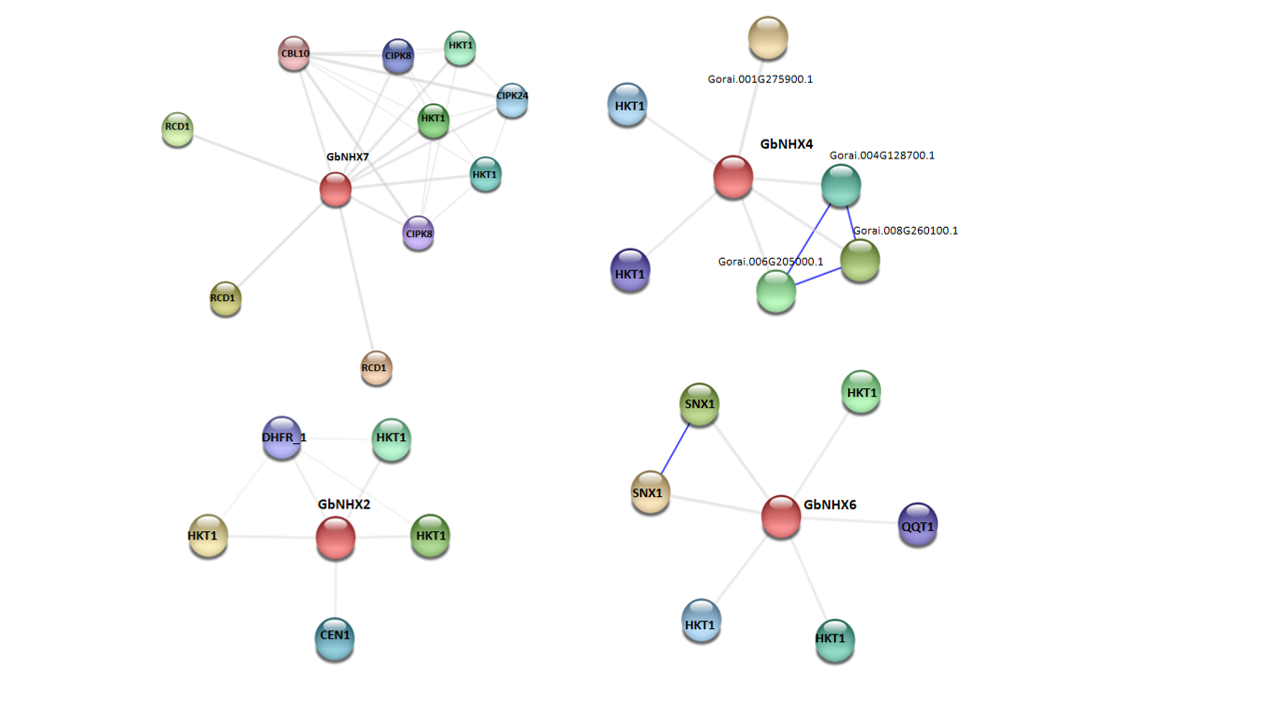

Supplement: Supplementary file 1 [file genes-11-00803-s001.zip › genes-850571 suppl/Figure S7 Sigle PPI.tif]

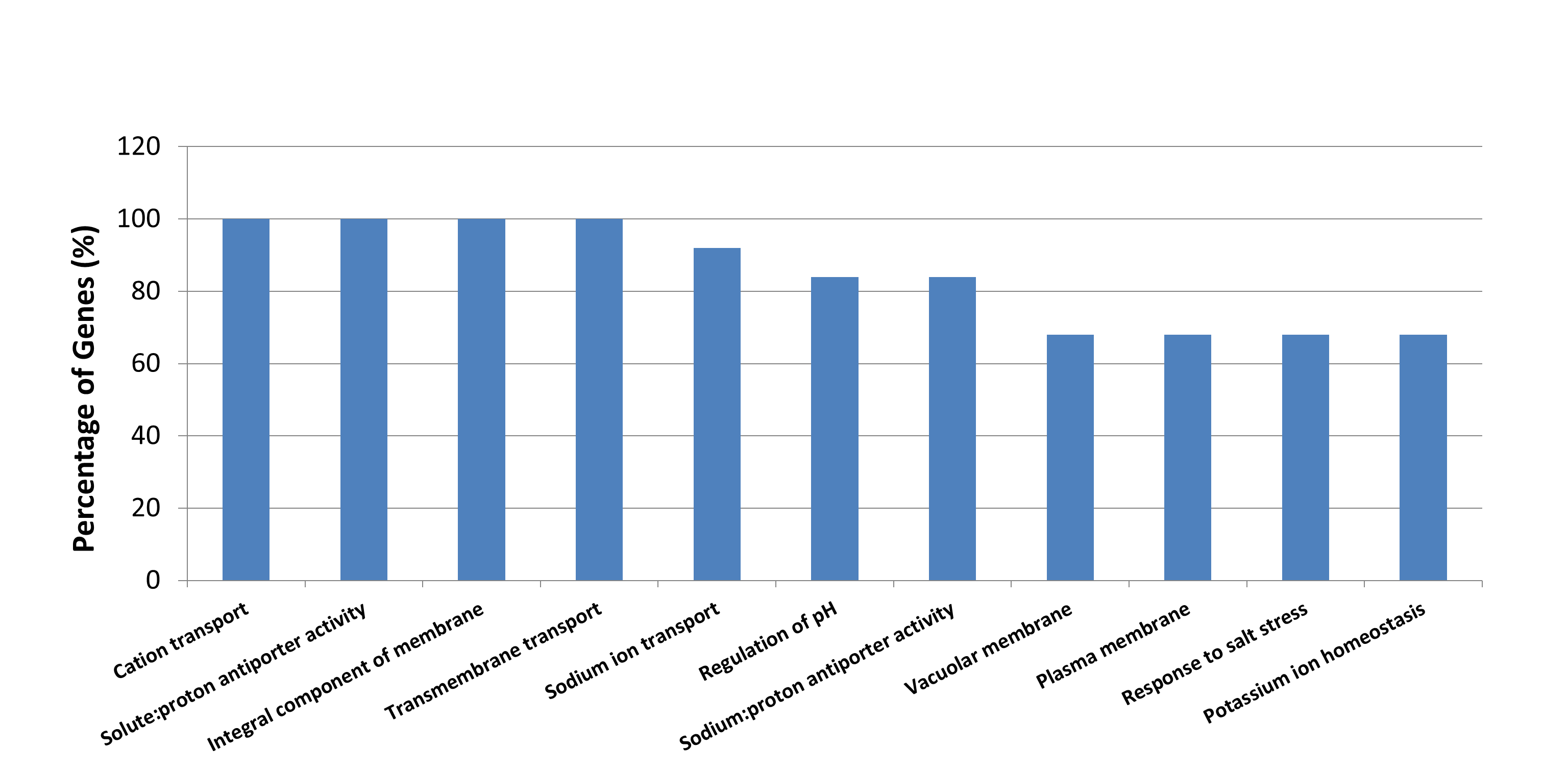

Supplement: Supplementary file 1 [file genes-11-00803-s001.zip › genes-850571 suppl/Figure S8 GO Terms.tif]
